# Hybrid Artificial Intelligence Approaches for Predicting Buckling Damage of Steel Columns Under Axial Compression

**DOI:** 10.3390/ma12101670

**Published:** 2019-05-22

**Authors:** Lu Minh Le, Hai-Bang Ly, Binh Thai Pham, Vuong Minh Le, Tuan Anh Pham, Duy-Hung Nguyen, Xuan-Tuan Tran, Tien-Thinh Le

**Affiliations:** 1 Faculty of Engineering, Vietnam National University of Agriculture, Gia Lam, Hanoi 100000, Vietnam; lmlu@vnua.edu.vn; 2 University of Transport Technology, Hanoi 100000, Vietnam; binhpt@utt.edu.vn (B.T.P.); anhpt@utt.edu.vn (T.A.P.); hungnd85@utt.edu.vn (D.-H.N.); tranxuantuank6xd@gmail.com (X.-T.T.); 3 NTT Hi-Tech Institute, Nguyen Tat Thanh University, Ho Chi Minh City 700000, Vietnam; 4 Institute of Research and Development, Duy Tan University, Da Nang 550000, Vietnam

**Keywords:** buckling behavior, Adaptive Neuro-Fuzzy Inference System, Particle Swarm Optimization, Genetic Algorithm, steel column

## Abstract

This study aims to investigate the prediction of critical buckling load of steel columns using two hybrid Artificial Intelligence (AI) models such as Adaptive Neuro-Fuzzy Inference System optimized by Genetic Algorithm (ANFIS-GA) and Adaptive Neuro-Fuzzy Inference System optimized by Particle Swarm Optimization (ANFIS-PSO). For this purpose, a total number of 57 experimental buckling tests of novel high strength steel Y-section columns were collected from the available literature to generate the dataset for training and validating the two proposed AI models. Quality assessment criteria such as coefficient of determination (R^2^), Mean Absolute Error (MAE) and Root Mean Squared Error (RMSE) were used to validate and evaluate the performance of the prediction models. Results showed that both ANFIS-GA and ANFIS-PSO had a strong ability in predicting the buckling load of steel columns, but ANFIS-PSO (R^2^ = 0.929, RMSE = 60.522 and MAE = 44.044) was slightly better than ANFIS-GA (R^2^ = 0.916, RMSE = 65.371 and MAE = 48.588). The two models were also robust even with the presence of input variability, as investigated via Monte Carlo simulations. This study showed that the hybrid AI techniques could help constructing an efficient numerical tool for buckling analysis.

## 1. Introduction

Instability is one of the most crucial failure of structural members under axial loading [1]. The problem of buckling was reported in the literature for structural components with different types of materials such as composite materials [2,3] or metallic shells [4]. The instability of such structural element depends on various parameters such as geometry of cross-section, length of structural components, boundary conditions, loads, etc. [5]. In order to characterize the instability behavior of structural components under compression, experimental investigations were carried out in many studies. In the work of Shi et al. [6], a laboratory experiment on stub steel structure under axial loading was conducted. Four specimens of square box section with similar slenderness ratio were prepared by welding 460 MPa steel plates together. The result indicated that the stability condition of steel tubes has been reduced regarding the standard designs. In many other experimental studies on buckling behavior of columns, such as box section (800 MPa yield strength steel) [7,8], I-section (690 and 460 MPa yield strength steels) [9,10] and hollow circular section (420 MPa yield strength steel) [11], test results demonstrated that buckling appeared earlier than estimation provided by existing design standards. However, all of these laboratory experiments were generally complex, costly and time consuming. In spite of all the efforts, it is not always possible to investigate a large number of variables such as length of columns, geometry of cross-section and mechanical properties of materials, as well as boundary conditions.

Experimental studies on buckling behavior of columns showed that the relationships between load and displacement is highly nonlinear [6,9]. Such nonlinear problem makes the analysis of structural elements behaviors under compression more complicated [12,13]. Theoretical works and semi-analytical analysis mainly concerned on cantilever beams [12,13,14] or simply supported beams [15,16]. With the development of numerical analysis, finite element method has been widely applied to investigate nonlinear phenomena in the field of mechanics, especially for buckling problem. Shi et al. [11] used ANSYS software (Version 15, Ansys Inc., Canonsburg, PA, USA, 2014 ) to simulate the mechanical behavior of circular steel tubes under axial compression. Finite element approach for studying the instability of structural compression elements has also been reported in many works, for instance, Shi et al. [6,8] for steel rectangular tubes, Yang et al. [10] for steel columns with I cross-section or Jiang et al. [17] for hollow circular tubes made of Nickel Titanium alloy. The use of commercial software such as ANSYS [18] or ABAQUS (Version 6.9, Dassault Systemes Simulia Corp, Providence, RI, USA, 2009) [19] mostly applied only to specific cases with limited variations of input parameters such as cross-section geometry, length, mechanical properties, loads, etc. In order to profoundly analyze the problem and perform high-performance parallel computing with large data, code development in programming languages is essential. However, nonlinear finite element method remains so challenging for researchers, particularly in terms of nonlinear algorithm implementation [20,21,22,23,24,25]. Therefore, it becomes clear that a more robust manner is required to better understand and predict the buckling behavior of structural elements under compression.

In the past few years, Artificial Intelligence (AI) models are widely used in mechanical engineering for predicting the properties and behaviors of structural elements [26]. Lakshmi et al. [27] developed an artificial neural network for predicting the mechanical properties in superplastic region of austenite stainless steel. Thankachan et al. [28] numerically investigated an AI model for the degradation of metallic material properties under presence of hydrogen. The prediction of effective mechanical properties of heterogeneous materials have also been studied using soft-computing techniques [29,30,31]. Various mechanical properties of material such as shear strength [32,33,34], tensile strength [35,36] and compressive strength [37,38,39,40,41,42] were investigated in the literature using data-driven algorithms. Several failure modes of structural elements have also been investigated with the help of AI approaches. For instance, fatigue of steel components was studied using neural network algorithm [43]. In the works of Tan et al. [44] and Padil et al. [45], damage in steel beams has been detected, located and quantified using a non-probabilistic artificial neural network model. Regarding structural members instability, buckling behavior of structural elements under axial loading was predicted using artificial neural network technique for various geometries such as shells [46,47], panels [48], beams of I-section [49] or elliptical section [35,50]. The prediction of column buckling under the presence of crack has also been reported in the work of Bilgehan [51] using an Adaptive Neuro-Fuzzy Inference System model. Thus far, studies involving AI models could strongly explain and predict the mechanical behavior of structural elements, particularly in terms of buckling capacity.

The main purpose of this paper is to investigate the ability to predict the buckling load of steel columns using two hybrid AI models such as Genetic Algorithm combined with Adaptive Neuro-Fuzzy Inference System (ANFIS-GA) and Particle Swarm Optimization combined with Adaptive Neuro-Fuzzy Inference System (ANFIS-PSO). The main difference between this work and previous studies is that this is the first time these hybrid AI models have been performed for predicting the buckling load of steel columns. Moreover, Monte Carlo simulations were applied to evaluate the robustness of both ANFIS-GA and ANFIS-PSO models, taking into account input variability. With this aim, 57 experimental results in the work of Yu et al. [52] with input variables (length of columns, geometry of cross-section, initial geometrical deviation in both x and y directions) and one output variable (the buckling load of 420 MPa high strength steel Y-section columns with slenderness ratio varying from 30 to 80) was used to generate training and testing datasets. Several statistical quality assessments such as the coefficient of determination (R^2^), Root Mean Squared Error (RMSE) and Mean Absolute Error (MAE) were used to validate the performance of the proposed models.

## 2. Methods Used

### 2.1. Machine Learning Methods

#### 2.1.1. Adaptive Networks-Based Fuzzy Inference System

A hybrid AI model constructed by coupling the Fuzzy Logic (FL) and Artificial Neural Networks (ANN) is well-known as Adaptive Networks-Based Fuzzy Inference System (ANFIS) [53]. In such a model, the number of nodes, which are connected by directional links in the ANN, can estimate the fuzzy parameters of fuzzy logic algorithm [54]. The main principle of the ANFIS lies in the construction of a set of fuzzy if-then rules with suitable membership functions to create the stipulated input and output variables [51]. More specifically, it is supposed that there are two input parameters (x and y) and one output variable (f), a base fuzzy if-then rules used in the ANFIS can be shown as follows [55]:

If *x* is A_1_ and *y* is B_1_, then *f*_1_*= a*_1_*x + b*_1_*y + r*_1_ (rule 1); (1)

If *x* is A_2_ and *y* is B_2_, then *f*_2_*= a*_2_*x + b*_2_*y + r*_2_ (rule 2). (2)

where *A*_1_, *A*_2_, *B*_1_, *B*_2_ are the functions of *x* and *y*, *p*_1_, *q*_1_, *r*_1_ and *p*_2_, *q*_2_, *r*_2_ are the linear output parameters of rule 1 and rule 2, respectively.

**Figure 1:**
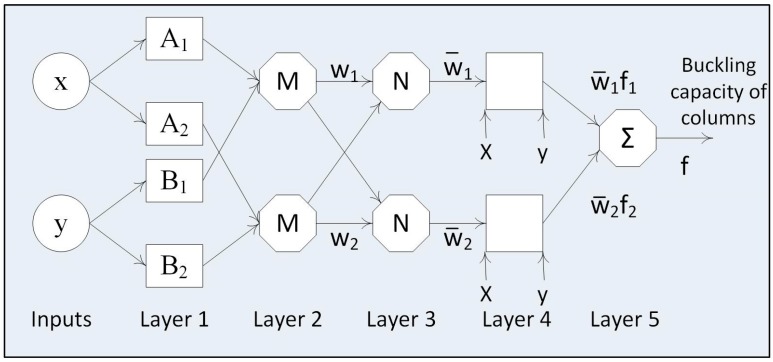
Architecture of Adaptive Neuro-Fuzzy Inference System (ANFIS) technique.

The structure of the ANFIS, which includes two inputs and one output (Figure 1), consists of five main layers, connected by adaptive nodes and fixed nodes as follow [53]:

**Layer 1:** Every node in this layer is a squared node with a node function as below:
  (3) $$O_{1,i}=\mu_{A}{}_{i}\left(x\right)$$
  (4) $$O_{1,i}=\mu_{B}{}_{i}\left(y\right)$$
  where $A_{i\left(x\right)}$ and $B_{i\left(y\right)}$ are linguistic labels of the inputs x and y, respectively, and $\mu_{A}{}_{i}\left(x\right)$ and $\mu_{B}{}_{i}\left(y\right)$ are the membership functions of $A_{i\left(x\right)}$ and $B_{i\left(y\right)}$.
**Layer 2:** Every node in this layer is fixed and labeled with an “*M*” sign, multiplies the incoming signals and sends the output.
  (5) $$O_{2,i}=w_{i}=\mu_{A}{}_{i}\left(x\right).\mu_{B}{}_{i}\left(y\right)$$
**Layer 3:** Every node is fixed and labeled with “*N*”. The outputs are normalized as:
  (6) $$O_{3,i}={\bar{w}}_{i}=\frac{w_{i}}{\sum_{j=1}^{2}w_{j}}$$
**Layer 4:** Every node in this layer is an adaptive node with the node function indicated as following equation:
  (7) $$O_{4,i}={\bar{w}}_{i}f_{i}=w_{i}\left(a_{i}x+b_{i}y+r_{i}\right)$$
  where $w_{i}$ infers the outputs of layer 3.
**Layer 5:** Every node in this layer is a single fixed node and labeled with “Σ”; it sums up all incoming signals to compute the overall output:
  (8) $$O_{5,i}=\underset{i}{\sum }{\bar{w}}_{i}f_{i}=\frac{\sum_{i}w_{i}f_{i}}{\sum_{i}w_{i}}$$

#### 2.1.2. Genetic Algorithm

Genetic Algorithm (GA) is a computational method inspired by the principle of biological evolution, which is used for optimization and searching. The idea of GA was first introduced by Holland and his students [56,57]. GA consists in moving a population of chromosomes (containing strings of ones and zeros, which are called genes) to a new population that outperforms the old one, using natural selection processes such as crossover or mutation [58,59].

**Figure 2:**
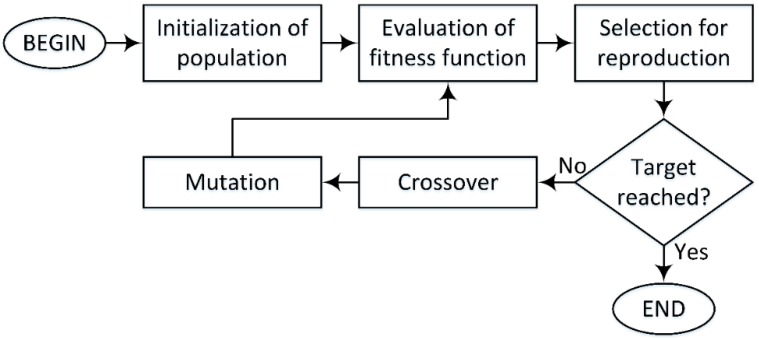
The structure of Genetic Algorithm.

Figure 2 illustrates the structure of GA, which can be presented in five main steps:

(1) Initialization of population: in this step, an initial set of solutions (population of chromosomes) to the current problem is introduced. Given *N* such that the size of the population (number of chromosomes), the choice of *N* is important, if *N* is too big, the algorithm might take too much time to perform. On the other hand, if *N* is too small, it might not be sufficient to reach an optimal solution. The choice of a fitness function is also defined in this step in order to evaluate how good the solution is in the next step.
(2) Evaluation of fitness function value: in this step, the fitness function value of each chromosome in the population is evaluated in order to verify if the chromosome is good enough to be reproduced.
(3) Selection for reproduction: in this step, a selection of best chromosomes is performed based on the fitness function value of each chromosome. The better they fit the fitness function, the more likely they will be chosen to be reproduced. After this step, if the stopping criterion is reached, the algorithm will stop; if not, the next two steps will be executed.
(4) Crossover: this step is realized with the contribution of two chromosomes. A crossover point is randomly chosen inside the chromosome; then, offspring are created by exchanging genes from their parents. For example, considering two chromosomes *S*_1_ and *S*_2_ defined as:
  (9) $$S_{1}=\left[1\;1\;1\;1\;1\right]$$
  (10) $$S_{2}=\left[0\;0\;0\;0\;0\right]$$
  Given the crossover point *i* = 2, we have the new offspring of *S*_1_ and *S*_2_ such that:
  (11) $$S_{1}^{\prime }=\left[0\;0\;1\;1\;1\right]$$
  (12) $$S_{2}^{\prime }=\left[1\;1\;0\;0\;0\right]$$
(5) Mutation: this process is performed within each individual offspring after crossover, their genes can be mutated in order to produce more offspring. For example, the new offspring of *S*_1′_ after mutation can be expressed such that:
  (13) $$S_{1}^{\prime \prime }=\left[1\;0\;1\;0\;1\right]$$

The diversity of the population can be maintained via the mutation process, which could also prevent the premature convergence. The GA technique has been successfully applied in various hybrid optimization studies. As typical examples, the GA was combined with both Support Vector Regression [60] and Response Surface Methodology [61] respectively in order to optimize friction welding process parameters for increasing tensile strength of ductile iron.

#### 2.1.3. Particle Swarm Optimization

Particle Swarm Optimization (PSO) is an optimization approach based on social behavior of animals such that the movement of organisms in a bird flock or a fish school, which is initially proposed in Eberhart and Kennedy [62]. The PSO has been largely applied to solve optimization problems [63,64,65]. Its main idea is to optimize a problem by iteratively moving a group of particles towards the best position in a given search space. Inspired by the social behavior of animals, five main principles of the PSO algorithm are outlined in the works of van den Bergh [66] and Wang et al. [67]:

- Proximity: it is able to perform simple calculations in time and space.
- Stability: the swarm does not change the behavior regarding every environment change.
- Quality: it is able to detect the quality change in the environment and respond to it.
- Diverse response: it has no limitation in the response to environment change.
- Adaptability: it is able to know if the change is worthy.

**Figure 3:**
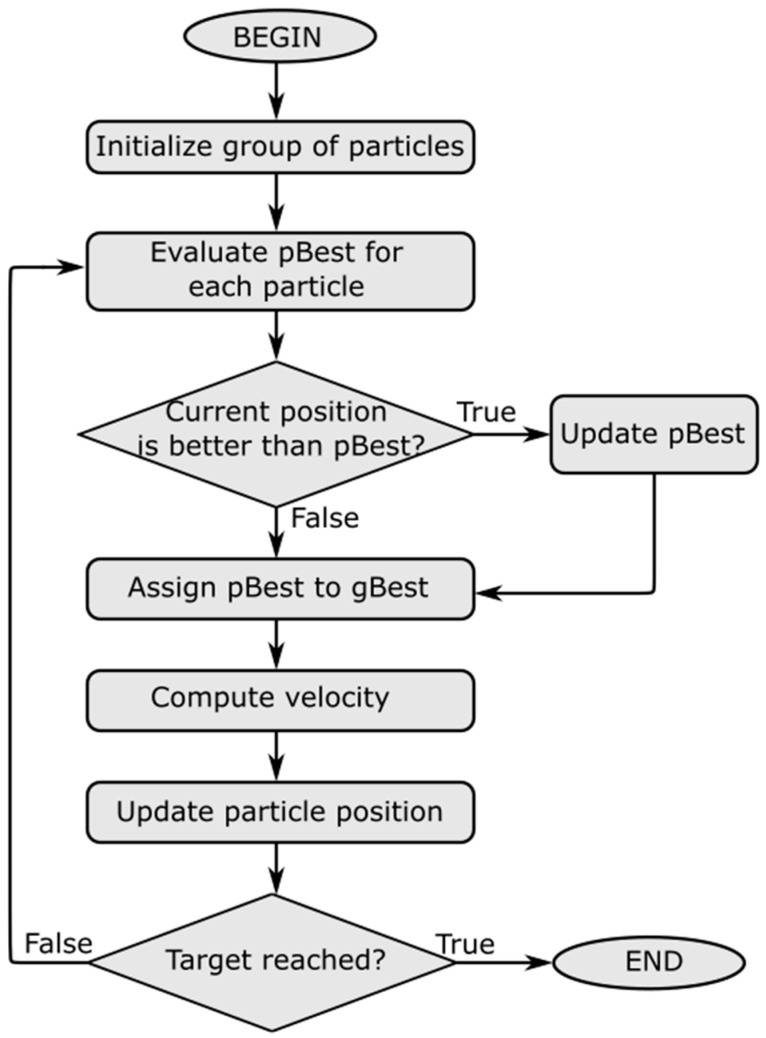
The Particle Swarm Optimization (PSO) algorithm.

There are two main operators in the PSO structure: position update and velocity update. Figure 3 illustrates the basic algorithm of PSO which consists of four mains steps at each iteration of the process:

(1) For each particle of the population, the best position that the particle has reached thus far, called pBest, is evaluated. If the current position is better than the previous position, then the particle position is updated; otherwise, the previous position is kept.
(2) Evaluate gBest, which is the best position of the particles in the entire population.
(3) Update the velocity using pBest and gBest. The new velocity is computed by:
  (14) $$v_{i}^{t+1}=v_{i}^{t}+\alpha \varepsilon_{1}\left[pBest_{i}^{t}-x_{i}^{t}\right]+\beta \varepsilon_{2}\left[gBest^{t}-x_{i}^{t}\right]$$
  where *i* is particle index, *t* is time index, ε_1_ and ε_2_ are two random vectors in range [0, 1] and *α* and *β* are positive constants.
(4) Update position of the particle. The new position of particles is calculated by:
  (15) $$x_{i}^{t+1}=x_{i}^{t}+v_{i}^{t+1}$$

These four steps are repeated to satisfy a stopping criterion, which means that the particles in the population are in the best-desired positions.

### 2.2. Validation Criteria

In this study, three statistical criteria namely coefficient of determination (R^2^), root mean squared error (RMSE) and mean absolute error (MAE) were introduced in order to validate the developed AI models. The R^2^ is widely used in regression analysis in order to estimate the percentage of variation of target data that could be achieved by predicted data [68]. Both RMSE and MAE measure the average magnitude of error [69]. However, RMSE is more useful in the case of large errors appeared (errors are squared using RMSE). All three criteria are important as they exhibit significant information one to another. R^2^, RMSE and MAE are defined by the following equations [70,71,72,73,74]:

(16) $$\mathrm{RMSE}=\sqrt{\frac{1}{N}\overset{N}{\underset{j=1}{\sum }}{\left(p_{0,j}{-p}_{t,j}\right)}^{2}}$$

(17) $$\mathrm{MAE}=\frac{1}{N}\overset{N}{\underset{j=1}{\sum }}\left|p_{0,j}{-p}_{t,j}\right|$$

(18) $$R^{2}=\frac{\sum_{j=1}^{N}\left(p_{0,j}-\bar{p_{0}}\right)\left(p_{t,j}-\bar{p_{t}}\right)}{\sqrt{\sum_{j=1}^{N}{\left(p_{0,j}-\bar{p_{0}}\right)}^{2}\sum_{j=1}^{N}{\left(p_{t,j}-\bar{p_{t}}\right)}^{2}}},$$

where *N* is the number of the observations, $p_{0}$ and $\bar{p_{0}}$ are measured and mean measured values, while $p_{t}$ and $\bar{p_{t}}$ are predicted and mean predicted values of buckling critical load, respectively (*j* = 1:*N*).

### 2.3. Monte Carlo Method

**Figure 4:**
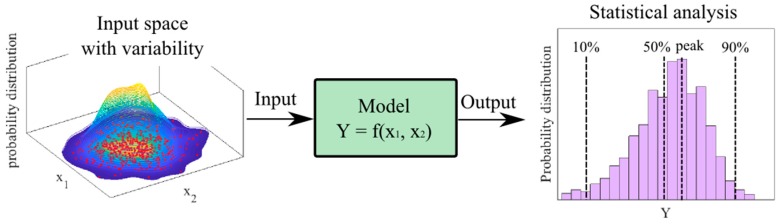
Monte Carlo method for propagating input variability to output results.

Monte Carlo method has been largely used in many domains of science, particularly in mechanical engineering [75]. Dao et al. [70] propagated random variability of various ingredients for the prediction of compressive strength of geopolymer concretes. Tensile deformation and failure process of composites were investigated by Yuan et al. [76] using Monte Carlo simulations. Guilleminot et al. [77] used Monte Carlo method in order to take into account the fluctuation in mechanical properties of random interphase in polymeric materials reinforced by nanoparticles. Various other uncertainty analyses have also been carried out thanks to the numerical solver such as contact mechanics [78], dynamical systems [79], measurement of mechanical properties [80] or viscoelastic composite structures [81]. This method has an ability to propagate variability of input variables to the output results by repeating randomly input sampling [82]. That way, automatic parallelization could be applied using Monte Carlo method to reduce computational time [83] without dropping significant statistical information in the input space. Figure 4 presents a schematization of using Monte Carlo method to propagate variability in the input space to output through models.

In this study, an indicator of convergence, called λ, was introduced in order to determine an optimal number of Monte Carlo runs, as defined by the following equation [84]:

(19) $$M\mapsto \lambda \left(M\right)=\frac{1}{U^{m}}\frac{1}{M}\overset{M}{\underset{i=1}{\sum }}U_{i},$$

where *M* is the number of Monte Carlo runs, *U* is the considered random variable and $U^{m}$ is the average value of *U*. Such convergence analysis could help optimize simulation time and also provide a reliable statistical result.

## 3. Data Used and Input Selection

**Figure 5:**
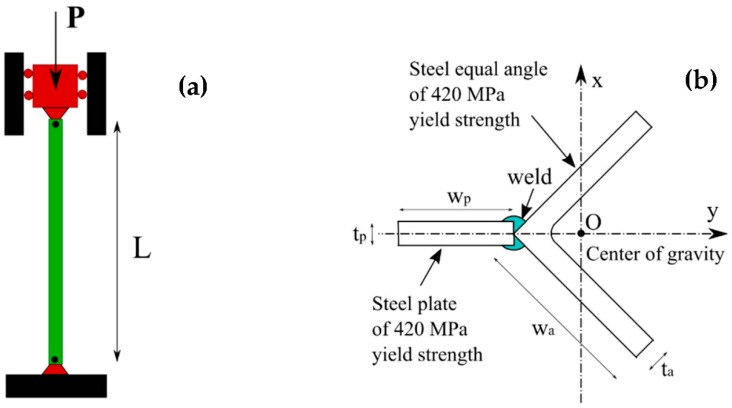
Configuration of columns: (**a**) pinned-pinned column under axial loading and (**b**) geometrical parameters of Y-section.

In this study, 57 experimental results on buckling behavior of Y-section steel column were extracted from the literature and summarized in Table 1. The dataset, conducted by Yu et al. [52], was achieved from measures of steel columns with pinned-pinned boundary conditions (Figure 5a). This form of cross-section has been recently used in transmission tower to increase the performance under various usage conditions. The Y-section columns were made by welding steel of equal angles and a steel plate (Figure 5b). Both components of the Y-section columns were manufactured from high strength steel of nominal 420 MPa yield strength and a Young’s modulus of 210 GPa. Along with the axial loading tests, initial geometrical imperfections such as bending at middle of the columns, loading eccentricity at both top and bottom cross-sections were also measured. The fluctuations of the real material properties compared to the nominal one varied from 450 to 496 MPa of yield strength, as observed by the authors. This finding has been reported for different high strength steels, such as circular tube section [11] or box section [6]. The residual stresses have also been measured in a longitudinal direction using blind hole drilling method [85]. It was shown that the cross-section could be self-balanced over the residual stresses (i.e., the resultant force and moment over the cross-section could be calculated as zero). Indeed, this is an important advantage of high strength over normal strength steel by reducing the ratio of residual stress to yield strength [6].

In order to construct the buckling prediction models of columns, only geometrical information and initial geometrical imperfections were considered as input parameters. The fluctuations of mechanical properties as well as the residual stresses were considered having no effect on buckling behavior of columns, as mentioned previously. However, initial geometrical imperfections could exhibit a significant impact on buckling behavior of Y-section columns and it is necessary to evaluate [86,87]. Geometrical information constituted of length of columns, width, thickness of steel equal angles and width and thickness of the steel plate. Initial geometrical imperfections were total deviations in the x and y directions. Summary of the dataset is indicated in Table 1, including additional information of buckling factor and slenderness ratios around the *x*-axis and *y*-axis. It is worth noticed that the slenderness ratios and the buckling factor are dependent parameters. It was observed that the length of columns varied from 925 mm to 3314 mm with mean value of 2003.86 mm and standard deviation of 636.51 mm. The width of steel equal angles ranged from 125 mm to 141.50 mm with mean value of 130.94 mm and standard deviation of 7.42 mm. The width of the steel plate varied from 60.00 mm to 101.20 mm with mean value of 81.46 mm and standard deviation of 16.69 mm. The thickness of the steel plate varied from 6.01 mm to 10.44 mm with mean value of 8.25 mm and standard deviation of 1.70 mm. The total deviation in x-direction ranged from −3.28‰ to 3.05‰ with mean value of 0.32‰ and standard deviation of 1.40‰. The total deviation in y-direction ranged from −2.82‰ to 3.51‰ with mean value of 0.81‰ and standard deviation of 1.61‰. The buckling load varied from 735 kN to 1631 kN with mean value of 1247.51 kN and standard deviation of 221.01 kN.

## 4. Methodology

**Figure 6:**
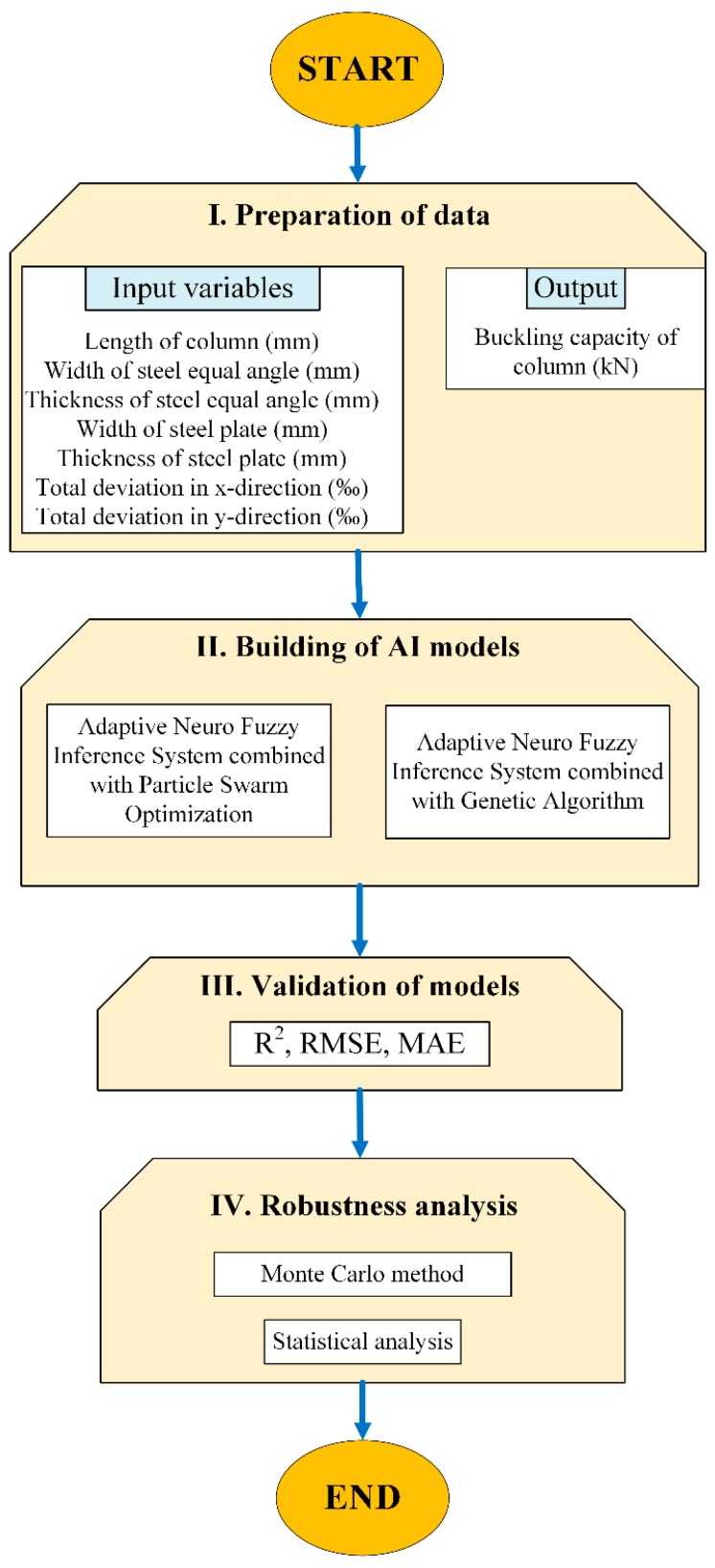
Methodology flow chart of this study.

The methodology of this study is presented in Figure 6, involving four main steps: (I) preparation of data, (II) building of AI models, (III) validation of the models and (IV) robustness analysis.

**Step I:** In this step, the data collected including seven input variables and one output was randomly split into training dataset, including 70% of data, and testing dataset with the remaining 30% of data.

**Step II:** The training dataset was used to train the AI models. Regarding the ANFIS-PSO, the PSO technique was performed using 25 particles, the inertia weight of 0.4 and 1000 iterations to optimize the consequent and antecedent parameters of the ANFIS model. Regarding ANFIS-GA model, the real coded GA technique was performed using a population size of 25. Such a number of population size was chosen after trial-and-error testing with respect to both dimensionality of the problem and computational time. Noting that population size in the range of 20 to 50 is commonly used in optimization problems involving GA technique, for instance in Almeida et al. [88], Valarmathi et al. [89] or Cheng et al. [90]. All GA parameters employed in this study are indicated in Table 2 as below:

**Step III:** The trained models were tested and validated using the testing dataset. Statistical criteria such as R^2^, RMSE and MAE were introduced to validate the developed models. A comparative study of prediction performance between two AI models was also carried out.

**Step IV:** Monte Carlo simulations were conducted in order to investigate the robustness of AI methods under the presence of input variability. Within a limit number of specimens, 200 Monte Carlo runs were performed with ANFIS-GA and ANFIS-PSO in order to compare statistical analysis results.

## 5. Results and Discussion

### 5.1. Validation of Models and Prediction Capability

**Figure 7:**
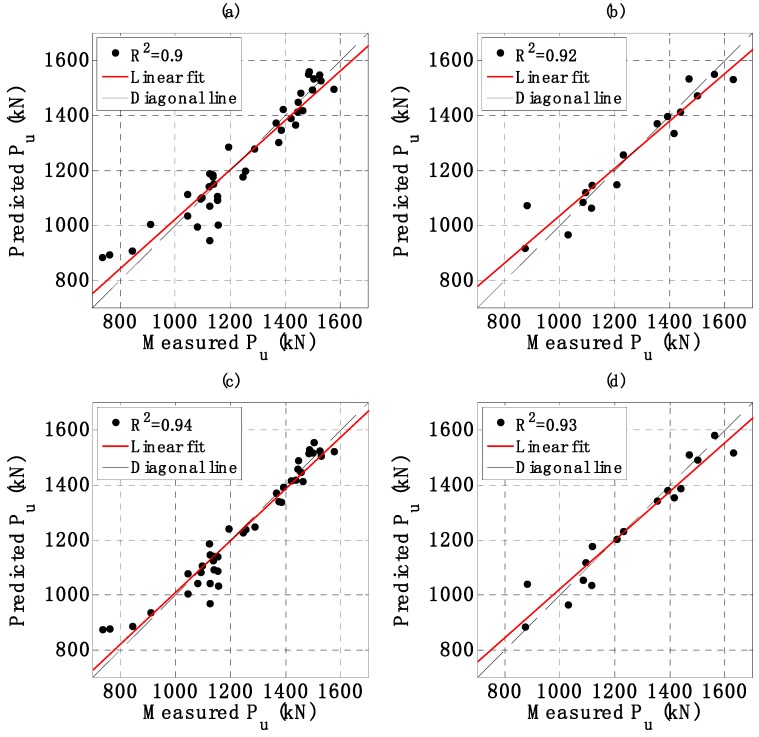
Regression results between measured Pu versus predicted Pu for the training part using (**a**) Adaptive Neuro-Fuzzy Inference System optimized by Genetic Algorithm (ANFIS-GA), (**c**) Adaptive Neuro-Fuzzy Inference System optimized by Particle Swarm Optimization (ANFIS-PSO); for the testing part using (**b**) ANFIS-GA, (**d**) ANFIS-PSO.

**Figure 8:**
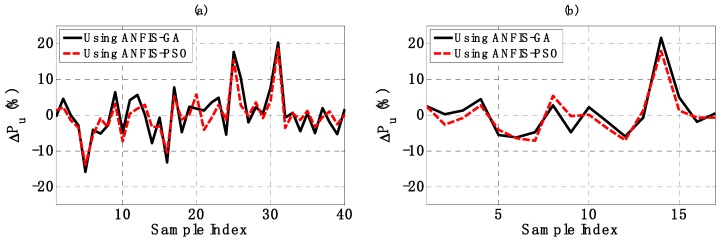
Graphs of ${\Delta P}_{u}$ in function of sample index for: (**a**) the training part; (**b**) the testing part.

In this section, a validation of the two proposed AI models is performed. The regression graphs between output results of AI models and the corresponding measured buckling capacity are shown in Figure 7a,c for the training dataset, Figure 7b,d for the testing dataset, using ANFIS-GA and ANFIS-PSO, respectively. A linear fit was also applied and plotted in each case. The normalized error ${\Delta P}_{u}$ was also introduced by the following equation:

(20) $${\Delta P}_{u}=\frac{P_{u}^{\mathrm{predicted}}{-P}_{u}^{\mathrm{measured}}}{P_{u}^{\mathrm{measured}}}\times 100,$$

where $P_{u}^{\mathrm{predicted}}$ and $P_{u}^{\mathrm{measured}}$ are values of predicted and measured *P_u_*, respectively. The variation of normalized error ${\Delta P}_{u}$ in function of sample index is shown in Figure 8a for the training part and in Figure 8b for the testing part. A strong correlation between predicted outputs of two AI models and experimental values of *P_u_* was observed, clearly demonstrating the effectiveness of these algorithms. Detailed values of R^2^, RMSE, MAE and ${\Delta P}_{u}$ are summarized in Table 3.

For the training part, ANFIS-PSO gave a higher value of R^2^ (0.937) compared to ANFIS-GA (0.899). In terms of RMSE and MAE, ANFIS-PSO (RMSE = 54.437 and MAE = 40.143) also presented a better performance compared to ANFIS-GA (RMSE = 68.711 and MAE = 53.824). Moreover, the mean value of normalized error ${\Delta P}_{u}^{m}$ of ANFIS-PSO is closer to zero than that of ANFIS-GA (${\Delta P}_{u}^{m}$ = 0.038 and 0.490 with ANFIS-PSO and ANFIS-GA, respectively). For comparison purpose, the standard deviation of normalized error ${\Delta P}_{u}^{\sigma }$ using ANFIS-PSO was 5.596, whereas ANFIS-GA was 6.832. This indicates that the predicted values by ANFIS-PSO had a low order of error fluctuation than that of ANFIS-GA model.

With regard to the testing part, ANFIS-PSO exhibited a better prediction capability than ANFIS-GA. Indeed, the values of R^2^, RMSE, MAE of ANFIS-PSO were 0.929, 60.522 and 44.044, while those of ANFIS-GA were 0.916, 65.371 and 48.588, respectively. On the other hand, the values of mean and standard deviation of the normalized error ${\Delta P}_{u}$ were −0.101, 5.844 for ANFIS-PSO and 0.540, 6.538 for ANFIS-GA, respectively.

Based on the computed values of R^2^, RMSE, MAE, ${\Delta P}_{u}^{m}$ and ${\Delta P}_{u}^{\sigma }$, it can be observed that ANFIS-PSO was a better model for predicting of column critical buckling load. However, ANFIS-GA also appeared a very promising candidate, as the difference between error criteria is rather small. Both ANFIS-PSO and ANFIS-GA were potential techniques that could be widely applied for predicting related problems in the field of mechanics.

### 5.2. Robustness of Models

**Figure 9:**
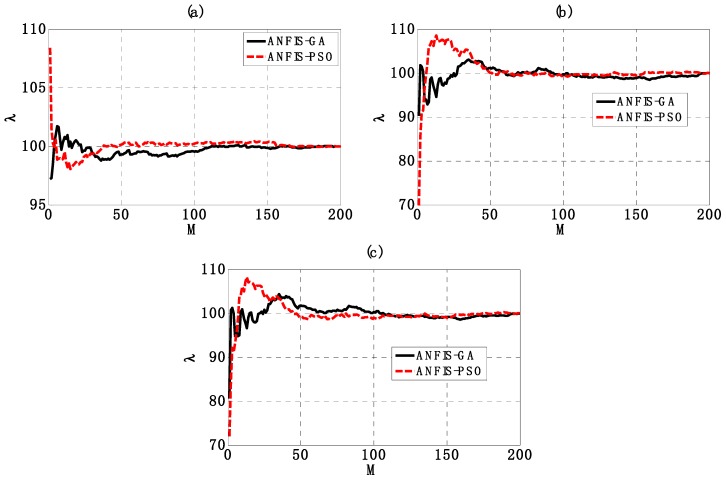
Graphs of the convergence indicator λ over 200 Monte Carlo realizations for (**a**) R^2^, (**b**) Root Mean Squared Error (RMSE) and (**c**) Mean Absolute Error (MAE).

The robustness of ANFIS-PSO and ANFIS-GA under the presence of input variability was investigated with the help of Monte Carlo simulations, as detailed in Section 2.3. Statistical analysis including convergence estimation (Equation (19)) of R^2^, RMSE and MAE distributions was carried out and summarized in Table 4. Figure 9a–c shows the statistical convergence of two AI models within 200 Monte Carlo simulations, in terms of R^2^, RMSE and MAE, respectively. The convergence indicator λ was deduced from the prediction outputs along with the number of Monte Carlo simulations M, as described in Equation (19). It is shown that the optimal number of Monte Carlo simulations (i.e., when the stationary solution is reached) was the same for both ANFIS-GA and ANFIS-PSO for R^2^, RMSE and MAE. Indeed, in the case of R^2^, the optimal number of Monte Carlo simulations is about M^opt^ = 170, whereas M^opt^ = 100 and 120 in the case of RMSE and MAE, respectively. It means that with respect to these criteria, a minimum number of Monte Carlo simulations of 170 was required to obtain reliable statistical analysis. Moreover, this indicates that the proposed number of Monte Carlo simulation in this study was sufficient.

**Figure 10:**
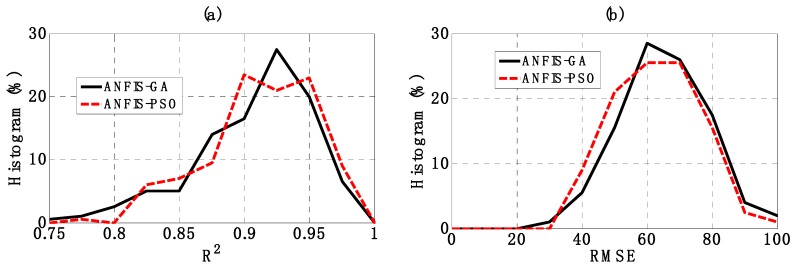

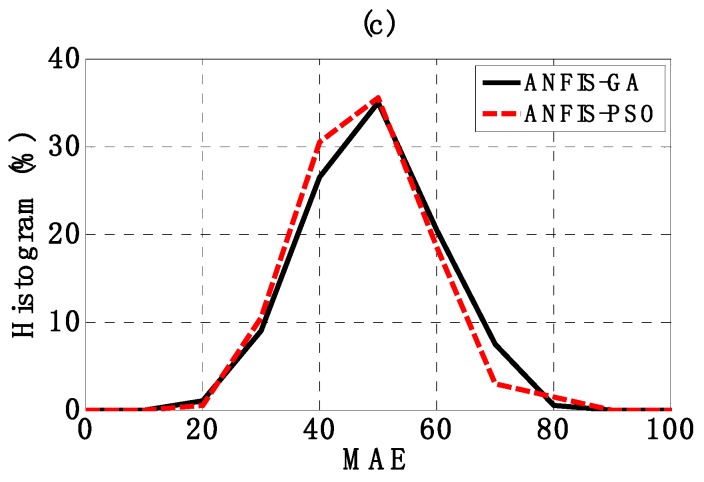
Graphs of the histogram of (**a**) R^2^, (**b**) RMSE and (**c**) MAE.

Figure 10 shows the histogram of R^2^, RMSE and MAE distributions, whereas statistical analysis is highlighted in Table 4. The values of average and standard deviation corresponding to the case of R^2^ were 0.905 and 0.051 for ANFIS-GA, whereas they were 0.910 and 0.047 for ANFIS-PSO. With respect to RMSE, these values were 65.247 and 13.199 for ANFIS-GA, 32.986 and 12.679 for ANFIS-PSO. In the case of MAE, the mean and standard deviation were 49.318 and 10.887 for ANFIS-GA, 47.629 and 10.343 for ANFIS-PSO. From the obtained results, it can be seen that the error distribution of ANFIS-GA is very close to that of ANFIS-PSO. However, ANFIS-PSO is slightly better than ANFIS-GA in terms of robustness.

From the statistical analysis, both ANFIS-GA and ANFIS-PSO produced excellent results in terms of robustness analysis. It could be concluded that hybrid AI approaches using evolutionary optimization algorithms such as PSO or GA combined with ANFIS are very promising computing models for predicting the buckling load of steel columns. Such models could supply efficient information that might be useful in the field of mechanics, civil engineering or related applications.

## 6. Conclusions

This work was devoted to the construction and validation of two hybrid AI approaches (ANFIS-GA and ANFIS-PSO) for predicting the critical buckling load of steel columns. To this aim, 57 measures of buckling load were collected from experiments in the literature for 420 MPa Y-section steel columns. The two AI models were constructed and validated using R^2^, RMSE and MAE criteria. Both ANFIS-GA and ANFIS-PSO models produced good results in predicting the buckling load of columns, but ANFIS-PSO (R^2^ = 0.929, RMSE = 60.522, MAE = 44.044) is slightly better than ANFIS-GA (R^2^ = 0.916, RMSE = 65.371, MAE = 48.588). ANFIS-GA and ANFIS-PSO were also proved as robust models under the variability of inputs using Monte Carlo method. However, in this study, several information was not considered in the modeling, such as the fluctuation of mechanical properties in terms of yield strength or longitudinal residual stresses. In addition, the AI models were constructed to predict only Y-section columns with various slenderness ratios (i.e., varying from 30 to 80). Therefore, these factors might be taken into account in further research, so that a better comprehension of instability of columns might be achieved, including empirical formula for linear buckling resistance analysis in order to facilitate the use in engineering applications. In addition, data normalization into a uniform range could also be helpful for minimizing bias within the datasets for better performance of the AI models. It is also suggested that numerical analysis involving AI techniques coupled with Finite Element method could be a potential candidate for establishing robust prediction models of structural element damage in the non-linear post-buckling regime.

## Figures and Tables

**Table 1:** Data used in this study [52] (2017, Elsevier Ltd.). *L* denotes the length of columns. Width and thickness of steel equal angles and steel plate are denoted by *w_a_*, *t_a_*, *w_p_* and *t_p_*, respectively, whereas *δ_x_* and *δ_y_* denote the total deviation in x and y-direction, respectively. *P_u_* is the buckling load of columns.

N°	Specimen	*L* (mm)	*w_a_* (mm)	*t_a_* (mm)	*w_p_* (mm)	*t_p_* (mm)	*δ_x_* (‰)	*δ_y_* (‰)	*P_u_* (kN)
1	M1130-1	1260	140.2	10.17	100.5	10.13	1.83	2.69	1523
2	M1130-2	1260	140.2	10.16	100.6	10.04	1.98	3.51	1483
3	M1130-3	1260	140.2	10.38	100.1	10.19	0.42	1.48	1631
-	-	-	-	-	-	-	-	-	-
-	-	-	-	-	-	-	-	-	-
55	M6680-1	2668	125.5	10.14	60.5	6.11	0.37	1.52	760
56	M6680-2	2674	125.8	10.41	60.6	6.2	0.2	−1.18	842
57	M6680-3	2672	125.5	10.09	60.2	6.16	1.73	1.41	735
	Min	925.00	125.00	10.00	60.00	6.01	−3.28	−2.82	735.00
	Average	2003.86	130.94	10.16	81.46	8.25	0.32	0.81	1247.51
	Max	3314.00	141.50	10.44	101.20	10.44	3.05	3.51	1631.00
	Standard deviation	636.51	7.42	0.11	16.69	1.70	1.40	1.61	221.01

**Table 2:** Parameters of Genetic Algorithm (GA) used in this study.

Parameters	Value
Population size	25
Length of chromosome	220
Fitness function	linear ranking
Cross-over type	random pair
Cross-over probability	0.4
Number of off-springs	10
Mutation type	random
Mutation probability	0.7
Number of mutants	18
Mutation rate	0.15
Selection function	fitness proportionate selection (roulette wheel selection)

**Table 3:** Summary information of prediction capability (${\Delta P}_{u}^{m}$ and ${\Delta P}_{u}^{\sigma }$ are the average and standard deviation of ${\Delta P}_{u}$, respectively).

Dataset	Methods	R^2^	RMSE (kN)	MAE (kN)	${\Delta P}_{u}^{m}(\%)$	${\Delta P}_{u}^{\sigma }(\%)$
Training	ANFIS-GA	0.899	68.711	53.824	0.490	6.832
	ANFIS-PSO	0.937	54.437	40.143	0.038	5.596
Testing	ANFIS-GA	0.916	65.371	48.588	0.540	6.538
	ANFIS-PSO	0.929	60.522	44.044	−0.101	5.844

**Table 4:** Summary information of robustness analysis.

Criteria	Methods	Average	StD	M^opt^
R^2^	ANFIS-GA	0.905	0.051	170
	ANFIS-PSO	0.910	0.047	170
RMSE	ANFIS-GA	65.247	13.199	100
	ANFIS-PSO	62.986	12.679	100
MAE	ANFIS-GA	49.318	10.887	120
	ANFIS-PSO	47.629	10.343	120
